# Compact dual-band antenna array for environmental monitoring systems

**DOI:** 10.1038/s41598-025-21634-x

**Published:** 2025-10-20

**Authors:** Bassant H. El Swiefy, Anwer S. Abd El-Hameed, Angie R. Eldamak, Hala ELsadek, Hadia M. El-Hennawy

**Affiliations:** 1 Micro Strip Department, Electronics Research Institute, Cairo, 12622 Egypt; 2 Electronics Department, Ain Shams University, Cairo, 11535 Egypt

**Keywords:** Electrical and electronic engineering, Environmental sciences, Engineering

## Abstract

A portable, high-resolution, ground-based synthetic aperture radar is essential for effective environmental monitoring. This paper presents a compact microstrip antenna array with dual bands and dual polarizations. This antenna will be integrated into a radar system to aid disaster mitigation efforts. The proposed antenna array consists of 1 × 4 driven elements for each of the three input/output ports; each driven element is coupled to four parasitic patches for bandwidth enhancement, with two shared parasitic patches between each pair of adjacent driven patches. Two of these ports support vertical polarization and operate in the X-band, achieving a bandwidth of 0.52 GHz. In contrast, the third port supports horizontal polarization and operates in the Ku-band, achieving a bandwidth of 0.67 GHz. The proposed design comprises four stacked layers: the lowest layer is dedicated to the feeding network, followed by a ground layer with rectangular apertures for excitation. On top of the ground layer, a third layer contains the driven patches, and the fourth layer holds the parasitic elements. Using two different substrates, the prospective antenna has a 67.2 mm × 71.1 mm size. The dielectric constant for ground plane layer is 3.48 with height of 0.762 mm while the driven and parasitic patches are on another substrate with dielectric constant of 2.2 for better radiation and height of 1.57 mm. In the X-band, the half-power beamwidth (HPBW) is 80° in the E-plane and 28.5° in the H-plane. In the Ku-band, the HPBW is 35.2° in the elevation plane and 15.6° in the horizontal plane. The antenna acquires gain of 11.17 dBi in the X-band and 14.22 dBi in the Ku-band which is suitable for environmental monitoring system applications.

## Introduction

In the last decade, the role of radar observations in civilian remote sensing and Earth observation has become increasingly important, leading to significant progress in synthetic aperture radar (SAR) technology^1^. Providing high-resolution images continuously and in all climate conditions has been achieved over 30 years using Synthetic Aperture Radar (SAR). It supports diverse applications, including earthly exploration, geoscience, environmental observing, climate research, 2-D and 3-D planning, change recognition, safety, and 4-D planning (space and time)^2^. SAR systems emit high-power electromagnetic pulses, capturing the reflections of the backscattered signal either from the same antenna (monostatic) or from a different one, either bi-static or multi-static. The object’s physical (geometry, roughness) and electrical (permittivity) properties influence the backscattered signal’s amplitude and phase. Longer wavelengths penetrate media more deeply, contributing more to the reflected signal^2^.

Common frequency bands used in SAR systems include KA, KU, X, C, S, L, and P bands^1^. SAR systems can be categorized into several types depending on their mounting platforms and intended applications, such as Airborne SAR, Spaceborne SAR, Ground-Based SAR (GB-SAR), and Maritime SAR^3^.

GB-SAR is a powerful tool for monitoring ground surface displacements, using radar to generate high-resolution images that detect even minute movements over time. GB-SAR’s key advantages include its all-weather operation and continuous real-time data collection. To form the synthetic aperture, horn antennas are moved along a metal bar, one for sending and the other for collecting signals, using a traditional GB-SAR system. However, the system’s weight and size pose significant transportation challenges. Despite these issues, GB-SAR’s rapid data acquisition is essential for assessing rapidly changing atmospheric conditions and monitoring vibrations in large structures, hence making the right decisions at the right times^4^.

Traditionally, GB-SAR antennas have employed slotted waveguide arrays, a well-established technology known for excellent polarization and side lobe control. Space applications need lightweight forms. However, waveguides are bulky and challenging to manufacture. Moreover, sharing a single aperture between two distinct arrays operating at vastly different frequencies is impractical without affecting the array’s scanning capabilities and performance^5^. GB-SAR antennas are typically costly, bulky, and heavy^6^. Thus, microstrip antennas may be more appropriate for achieving lightweight and low-profile antennas for GB-SAR applications^5^.Thus, there is a strong need to minimize the antennas’ complexity, size, mass, and cost while ensuring multiband operation, dual-polarization, and high gain. Integrating two patch sizes within a single aperture can facilitate dual-band operation which is called shared-aperture GB-SAR^7^.

While shared-aperture designs have been widely implemented to minimize antenna size and maintain functionality, designing the feed networks remains a significant challenge. With these shared-aperture configurations, GB-SAR antennas can achieve high gain and excellent port isolation across multiple frequency bands. One method for designing shared-aperture DBDP antennas is to employ the perforated structures^8–10^. For example, involves using patches with perforated ones for antennas to work at low and high frequencies^11^. Combining print dipoles work at low frequency with antennas working at high frequency is another way^12^. Enhancing the impedance bandwidth to over 20%, dual-band, dual-polarized antennas are designed using multiple rectangular patches, with parasitic patches and slot-coupled feeds. Furthermore, two circular parasitic patches with a comparable feed configuration are incorporated to enable dual-band operation, achieving impedance bandwidths of 17% and 28%. The orthogonal configuration of the two feed networks allows for dual polarization, effectively reducing the number of active ports to only two^13^.Another way is an interlaced layout is also introduced in^14^. A multi-frequency polarized GB-SAR antenna is needed, with each feed network dedicated to a single polarization per frequency band. This results in high complexity, bulkiness, mass, and fabrication costs. A DBDP high-gain antenna based on the concept of Fabry–Perot resonant cavity is designed. This antenna array 8 × 8 units operates in both C and X bands with size of 140 mm × 140 mm to achieve high gain but with huge size and more complexity in feeding network^7^.

To realize high gain, several basic methods could be integrated gain with planar antennas, making them a viable alternative to the horn antennas and can be used for GB-SAR systems^15^. Antenna arrays generally consist of individual elements linked by a feeding network^16^. Effective control of the array’s radiation properties requires careful design of both the feeding network and the arrangement of the array elements. Additionally, using mutli-layer structures and metallic vias, can enhance patch antenna bandwidth. However, combining different design aspects and requirements in one structure need to be clarified and compromise the reliability of the antenna design^17,18^.

For the targeted application, antennas should be operating at two distinct bands. This could be achieved by using only one feeding port as in^19,20^, or by using two separate feeding ports for the two bands^21,22^. To expand the operational capabilities of SAR systems, a band that supports dual frequencies and incorporates dual-polarization antennas can be utilized. In^17^, a slot array comprising 25 elements operates in the K-band with a compact size of 108.5 × 18 mm^2^, achieving a gain of approximately 14.2 dBi and a bandwidth of 1.6 GHz. In comparison^23^, presents a 4 × 4 array functioning in the X-band, offering the same gain with a significantly larger size of 110 × 122 mm and a narrower bandwidth of around 0.128 GHz. The design in^24^, featuring the largest size of 300 × 300 mm with 40 units, operates across triple bands—X, S, and C—delivering a wide bandwidth of approximately 9.3 GHz and gain ranging from 12 to 25 dBi. Meanwhile^25^, demonstrates a much smaller design, measuring just 19.5 × 8 mm, with a gain of 8.5 dBi.

This paper primary contribution extends beyond compactness, demonstrating the effective integration of dual-band operation (X-band and Ku-band), polarization diversity, and enhanced radiation performance for GB-SAR use.This array features a low profile and strong separation across three ports within a compact spacing. The array comprises shared-aperture dual-band (X band and KU band) operation, with elements based on Nasimuddin’s model^26–29^, incorporating four parasitic patches. The proposed design includes three stacked dielectric substrates, parasitic patches, driven patches, ground planes, and feeding networks in both Ku-band and X-band. Dual-band functionality is achieved through the use of distinct feed networks and polarization diversity. Specifically, the X-band operates via two ports utilizing vertically polarized elements arranged to optimize spatial utilization, while the Ku-band is accessed through a third port employing horizontally polarized elements. This orthogonal polarization, combined with the higher frequency of the Ku-band, enables a narrower beam width and higher gain. Additionally, the integration of stacked parasitic patches and carefully chosen substrate materials allows both frequency bands to operate efficiently within the same footprint. These design strategies ensure independent operation of both bands with minimal mutual interference, while maintaining high aperture efficiency. The Ku-band and X-band bandwidths are demonstrated to be 0.66 GHz and 0.5 GHz, respectively. Isolation greater than 20 dB is achieved among the three ports. The total size of the array is 67.2 mm × 71.2 mm × 3.9 mm. The main lobe beam width measures 80° in the E-plane at low frequencies. In the H-plane, the main lobe beam width is 28.5°. At the Ku band, the E-plane main lobe beam width is 35.2°, while it is 15.6° in the H-plane. The rest of this manuscript is prepared as follows: Section II represents the steps of the final design of the array and simulated results. Section III provides fabricated design and measurement results, and Section V gives conclusions given in Section VIII.

## Design geometry

The design process started with three key steps. First, a single element was designed to operate in the X and Ku bands. Next, parasitic elements are added to the proposed design. Finally, the elements were arranged into a 1 × 4 array.

### Sub-element design

#### Ku band Element

**Fig.1:**
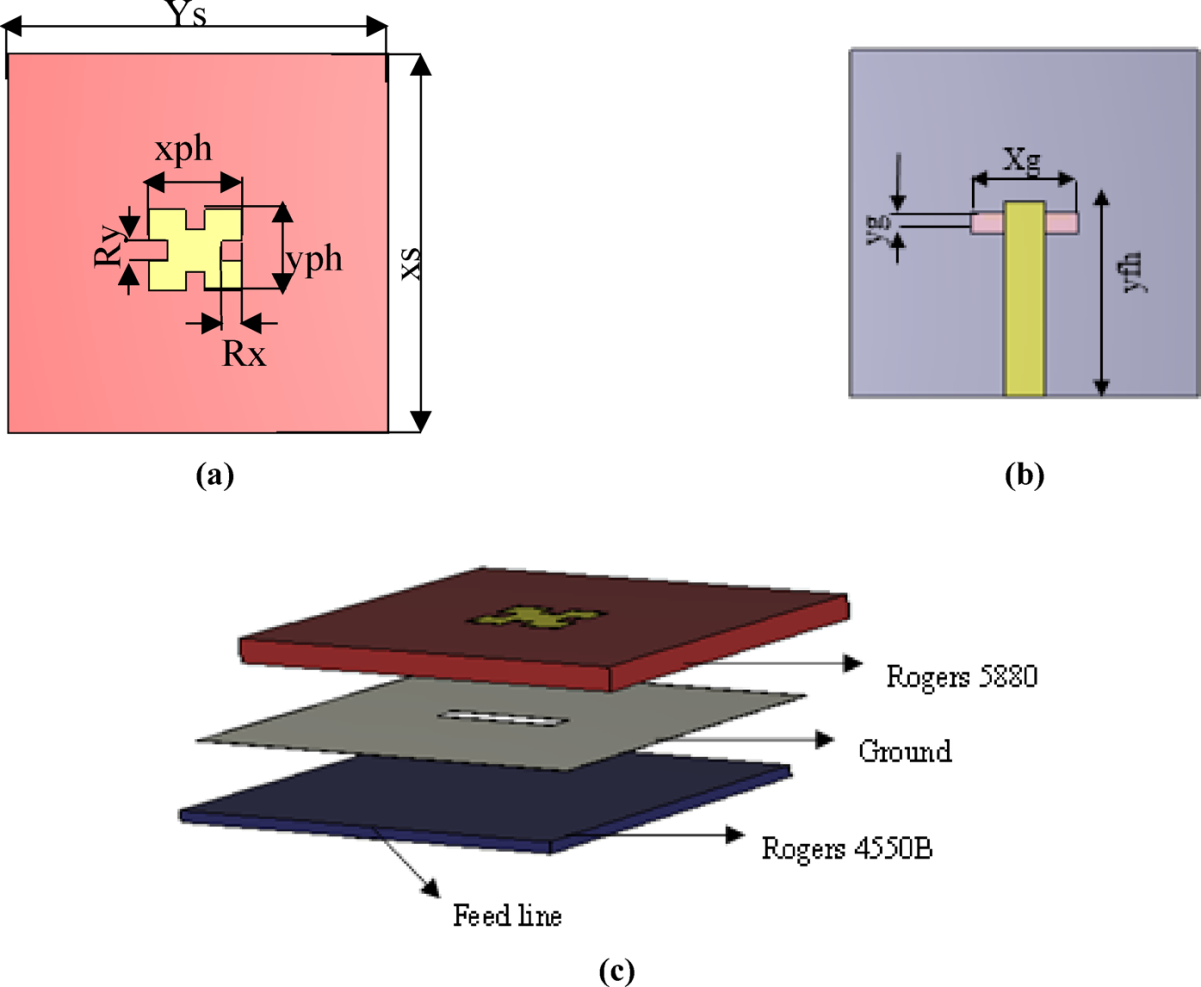
Single element works at Ku band. (**a**) Top substrate. (**b**) Back of 2nd substrate. (**c**) 3D design of single element.

The conventional type of microstrip planar antenna is a rectangular patch due to its simplicity and reliable performance. The rectangular patch can be conceptually viewed as a two-aperture radiator with a λg/2 spacing between its effective radiating edges, supporting the fundamental TM10 mode. To enable miniaturization without altering the aperture separation, four identical square slots are etched at the patch perimeter. These slots act as reactive LC loads, compelling the surface current to follow a longer path, effectively increasing the electrical length. This slow-wave effect reduces the resonant frequency while maintaining the same outer dimensions, resulting in a compact dual-band antenna^30^. However, a patch antenna typically offers a narrow bandwidth, unsuitable for radar systems. Several techniques can enhance bandwidth, such as aperture feeding, which significantly increases bandwidth. Aperture feeding consists of two substrates: a ground plane, a radiating patch, and a microstrip feed line. In this design, as shown in Fig. 1a,b, and c, a folded rectangular patch is placed on a top substrate of Rogers 5880 with a permittivity of 2.2 and a height of 1.57 mm. The feed is located on a bottom substrate of Rogers 4350B with a permittivity of 3.48 and a height of 0.762 mm. These two substrates are separated by a ground plane with a rectangular slot (aperture feed), shielding the antenna on the top substrate.

**Fig. 2:**
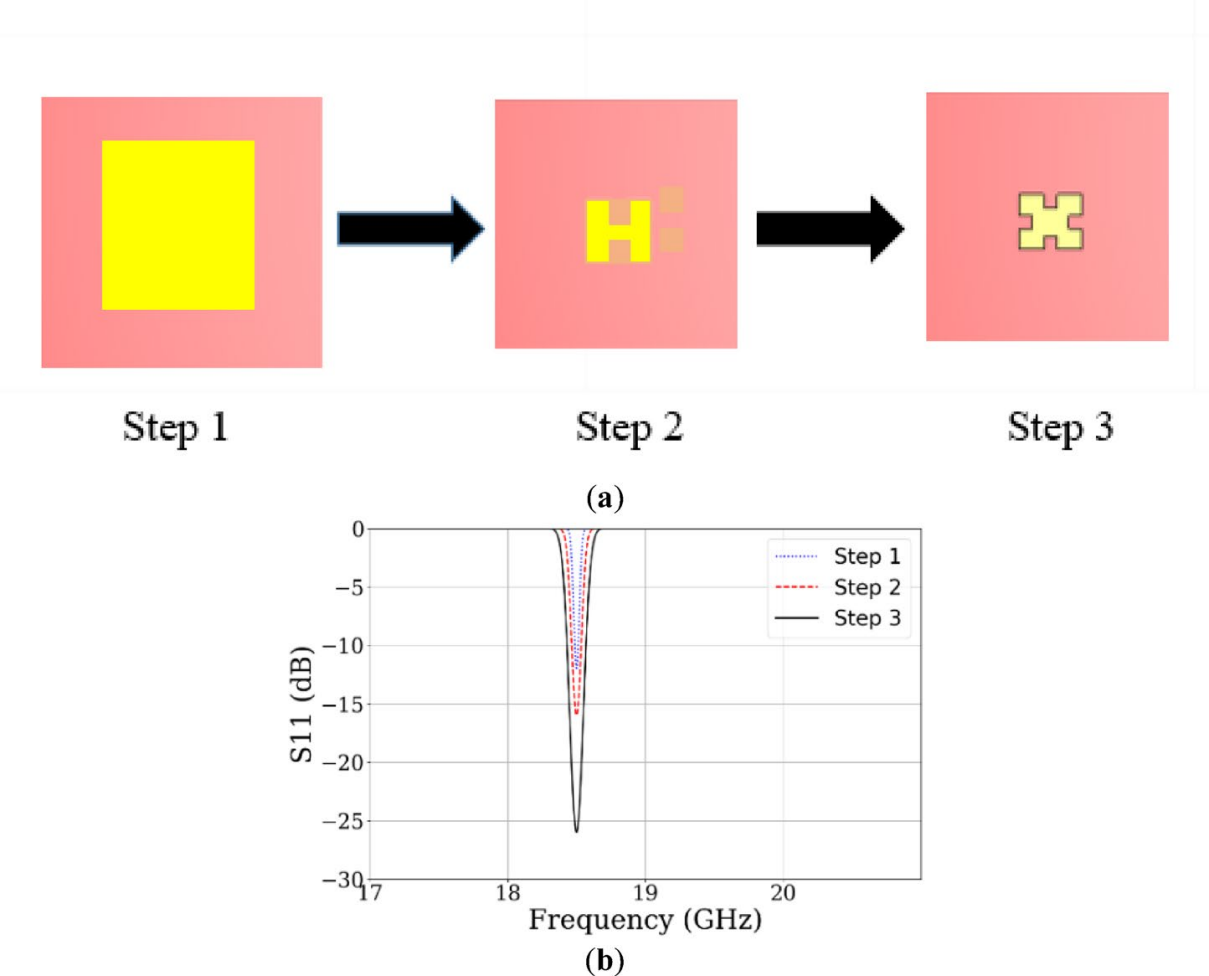
(**a**) Design steps of Ku-band element. (**b**) Simulated reflection coefficients of the design steps.

To systematically arrive at the optimal geometry of the Ku-band antenna element, a progressive design methodology is adopted through a multi-step design process as shown in Fig. 2a. First, the design begins with a conventional rectangular microstrip patch antenna, which initially exhibits poor impedance matching. To improve performance, two slots are introduced at the center of the horizontal edges of the patch, resulting in enhanced matching characteristics and reducing antenna size. Subsequently, two more square slots are added to the vertical edges to further reduce the antenna profile. The complete sequence of these three design steps leads to the final shape. Figure 2b shows the simulated reflection coefficients of all steps.

**Fig. 3:**
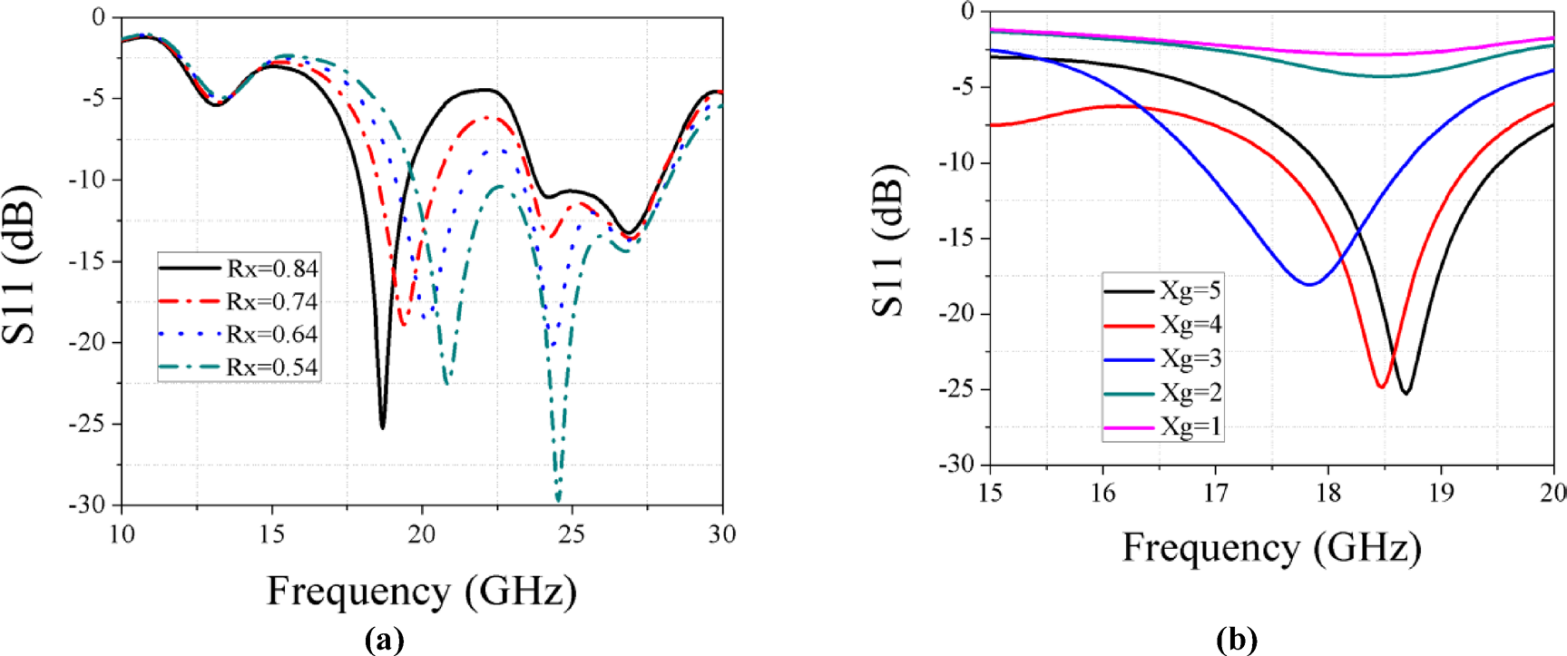
(**a**) Parametric analysis on Rx at Ku band. (**b**) Parametric analysis on Xg at Ku band.

Impedance matching was achieved by tuning the feed-stub length, aperture slot dimensions, and ground-slot width. Varying the length of the four edge-centered square slots from 0.64 to 1.04 mm revealed that a length of 0.84 mm provides optimal matching, as shown in Fig. 3a. Additionally, a parametric sweep of the ground-plane slot width (Xg) from 1 to 5 mm identified 5 mm as the best value, as illustrated in Fig. 3b. These optimized parameters were adopted in the final antenna design.

**Fig. 4:**
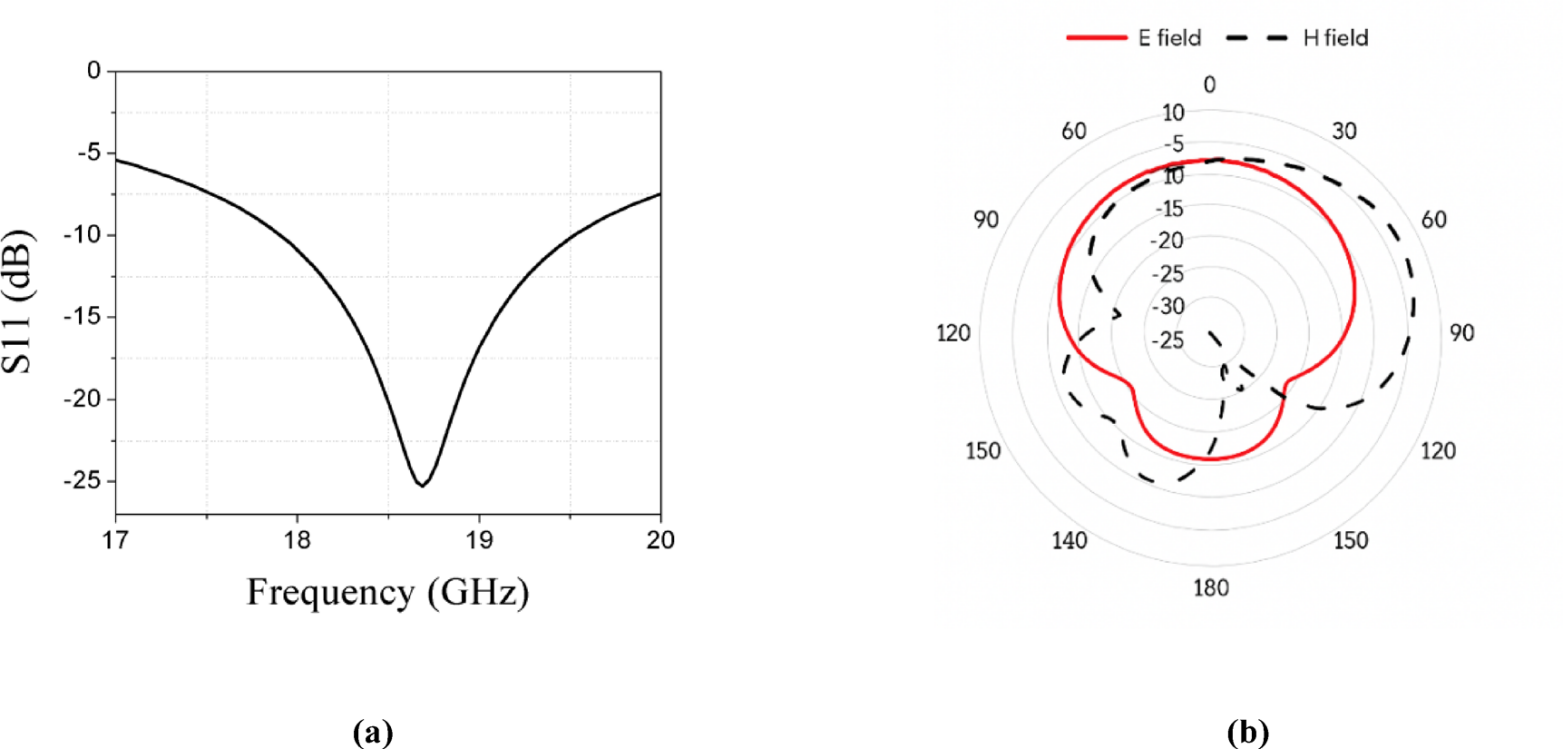
(**a**) Simulated results of S11 at Ku band. (**b**) Simulated E and H field patterns at Ku band.

This configuration achieves a bandwidth for the Ku band ranging from 17.9 to 19.5 GHz, as explained in Fig. 4a. Antenna achieves a gain of 8.12 dBi for the stated band. In Fig. 4b, the simulated radiation pattern at the Ku band is illustrated.

##### X band Element

**Fig. 5:**
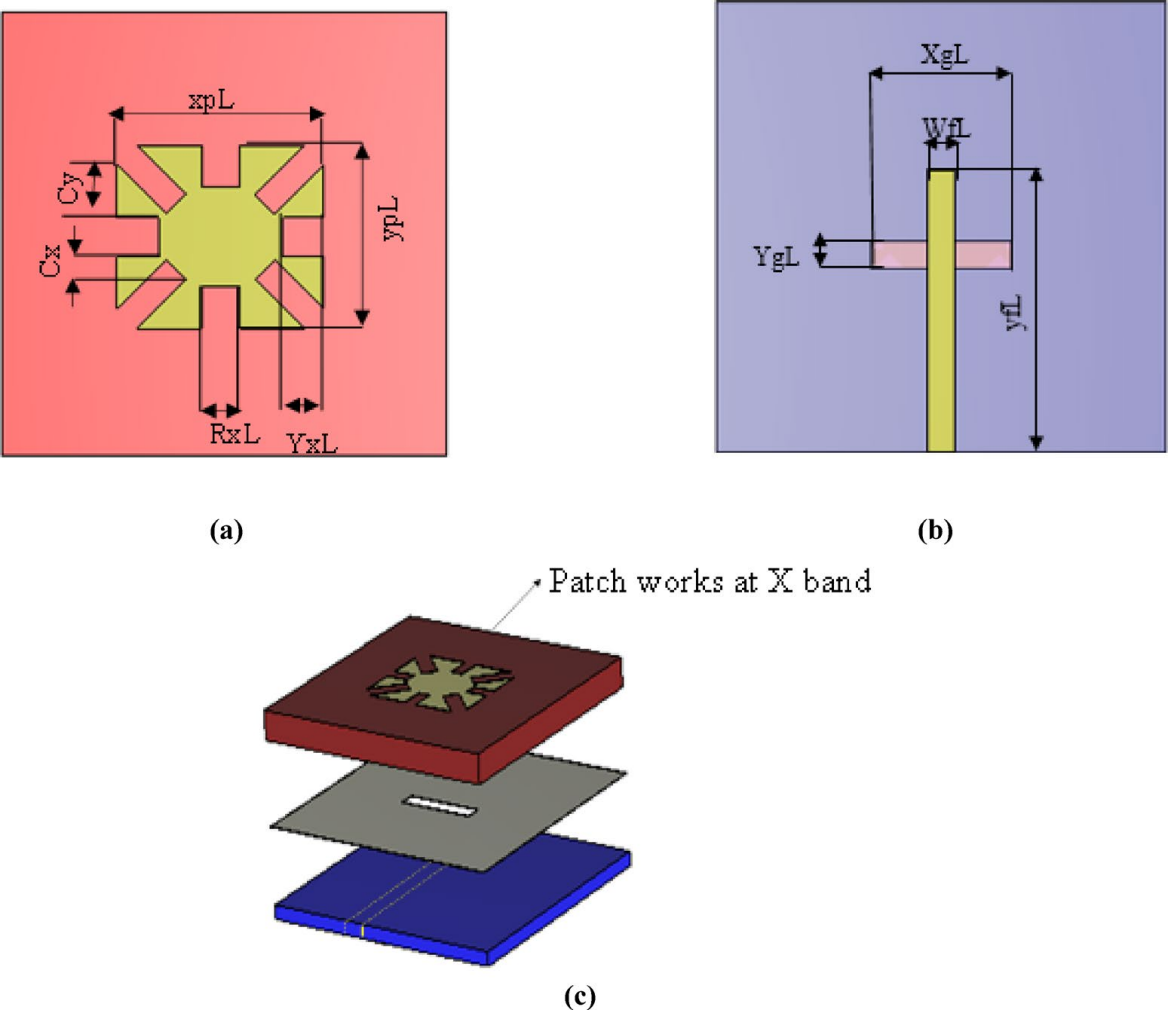
Single element works at X band. (**a**) Top substrate. (**b**) Back of 2nd substrate. (**c**) 3D design of single element.

**Fig. 6:**
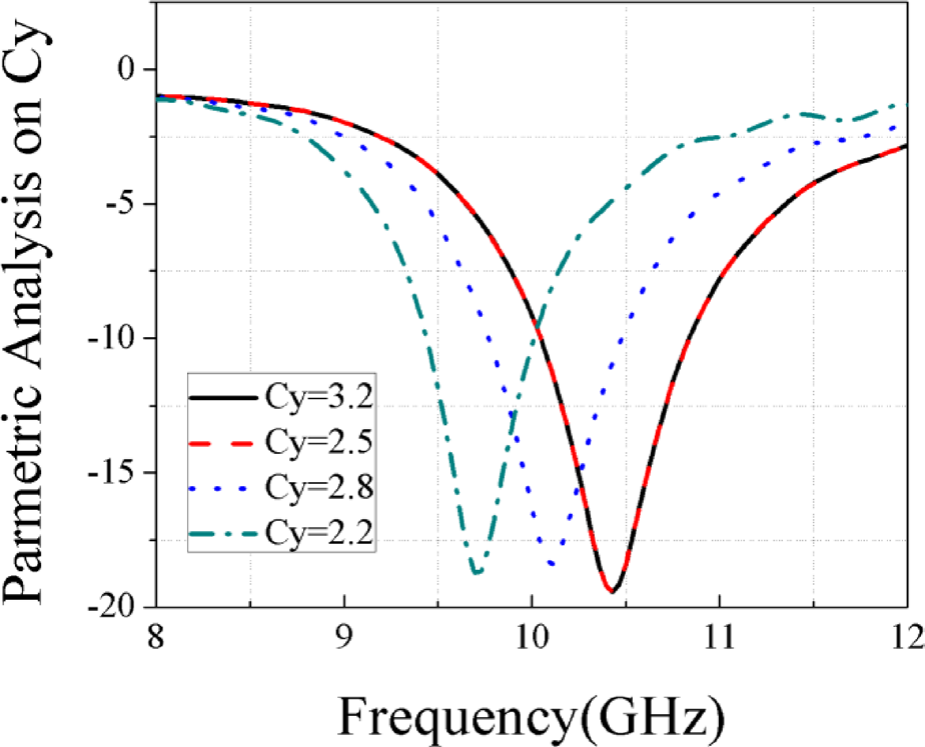
Parametric analysis on Cy at X band.

To adapt the design for the X-band, the Ku-band patch was scaled up by increasing its dimensions, as shown in Fig. 5a,b, and c. An additional rectangle was subtracted from each corner of the main patch to reduce its size. The optimized antenna dimensions are Cy = 2.5 mm as illustrated in Fig. 6.

**Fig. 7:**
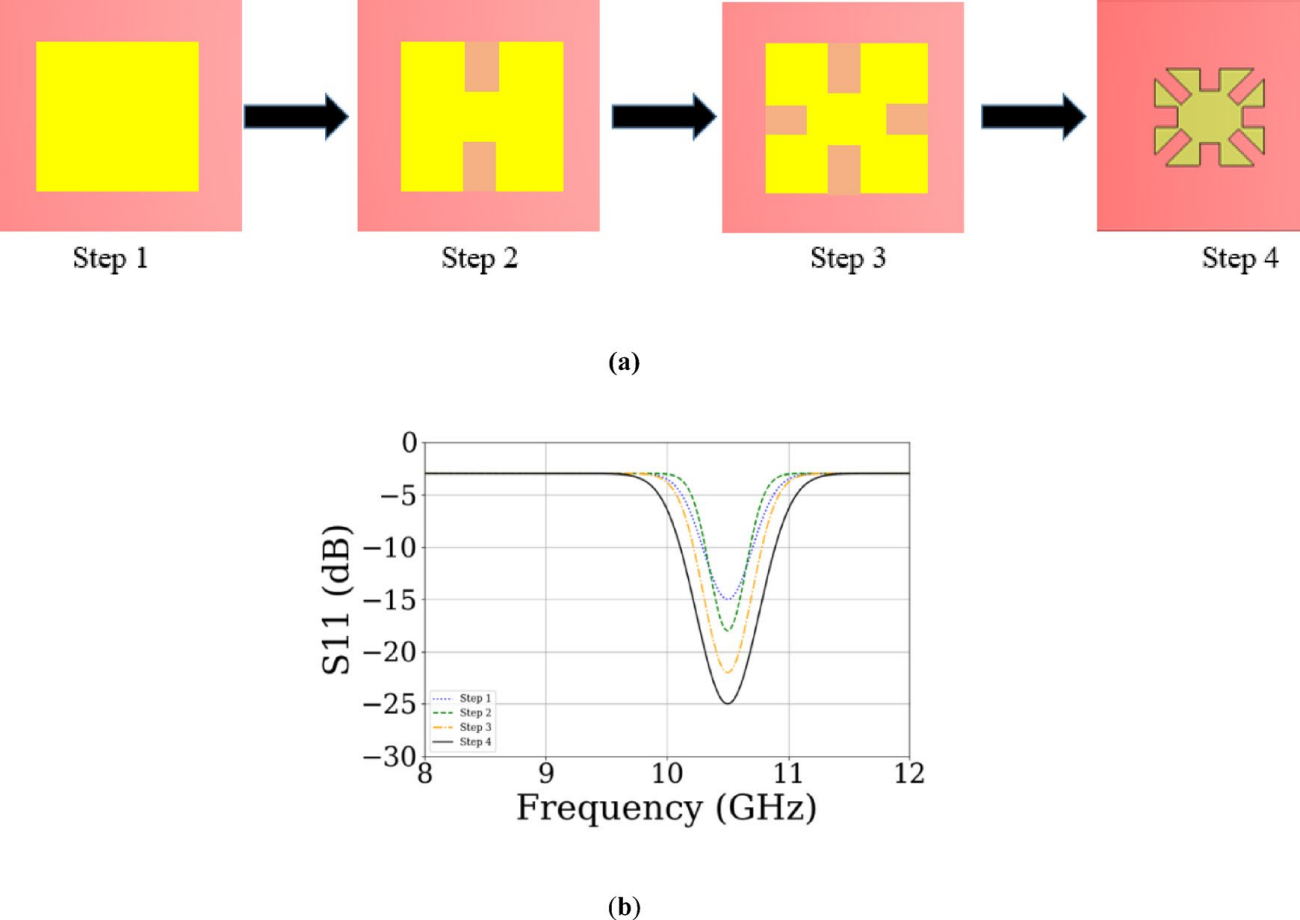
(**a**) Design steps of single X-band antenna element. (**b**) Simulated S₁₁ results at each step.

**Fig. 8:**
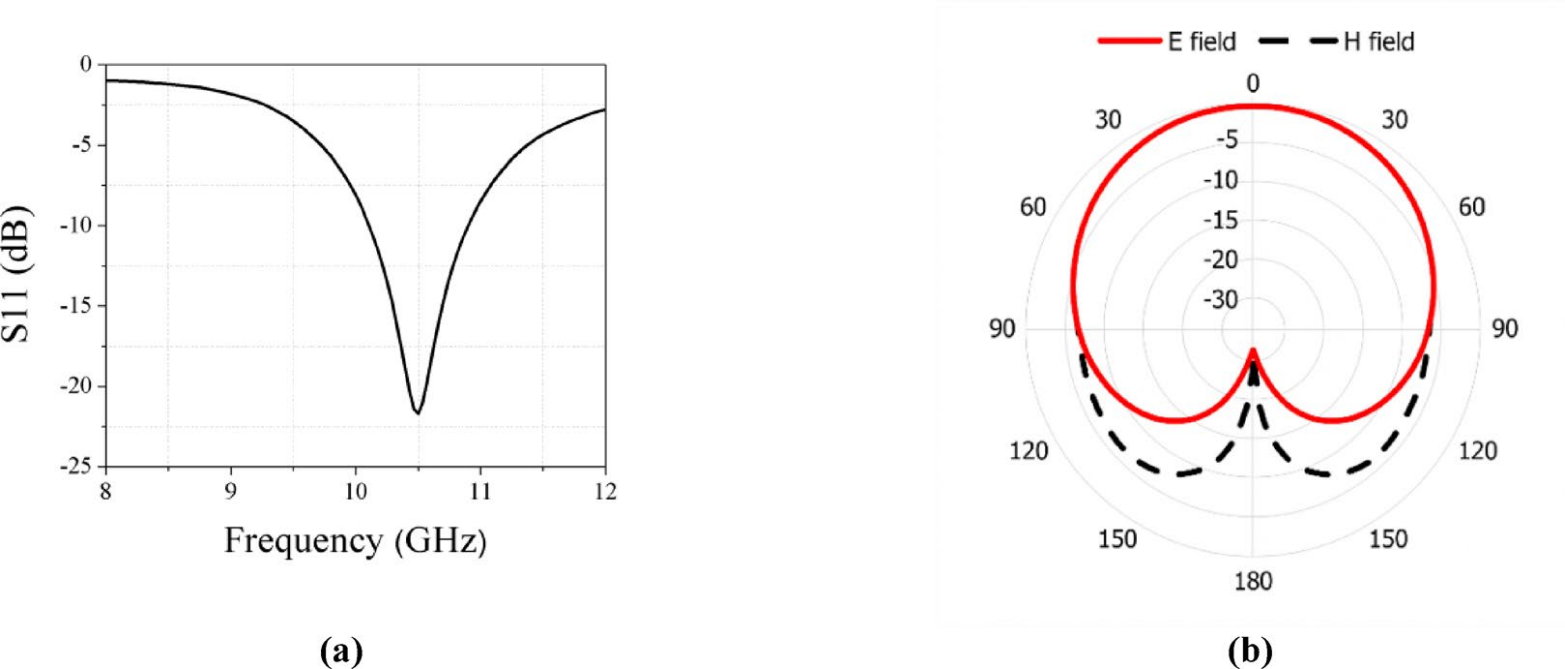
(**a**) Simulated results of S11 at X band. (**b**) Simulated E and H fields patterns at X band.

The ultimate shape of the X-band single-element antenna is obtained through a step-by-step design process similar to the Ku-band element, with an additional final stage for optimization, as shown in Fig. 7a. In the last step, corner sections are removed to enhance performance and further reduce size. The simulated reflection coefficients are presented in Fig. 7b. The resulting bandwidth in the X band is approximately 0.61 GHz, demonstrating good impedance matching at 10.5 GHz as illustrated in Fig. 8a. A gain of 4.862 dBi is achieved. Figure 8b presents the simulated radiation pattern.

### MIMO configuration

**Fig. 9:**
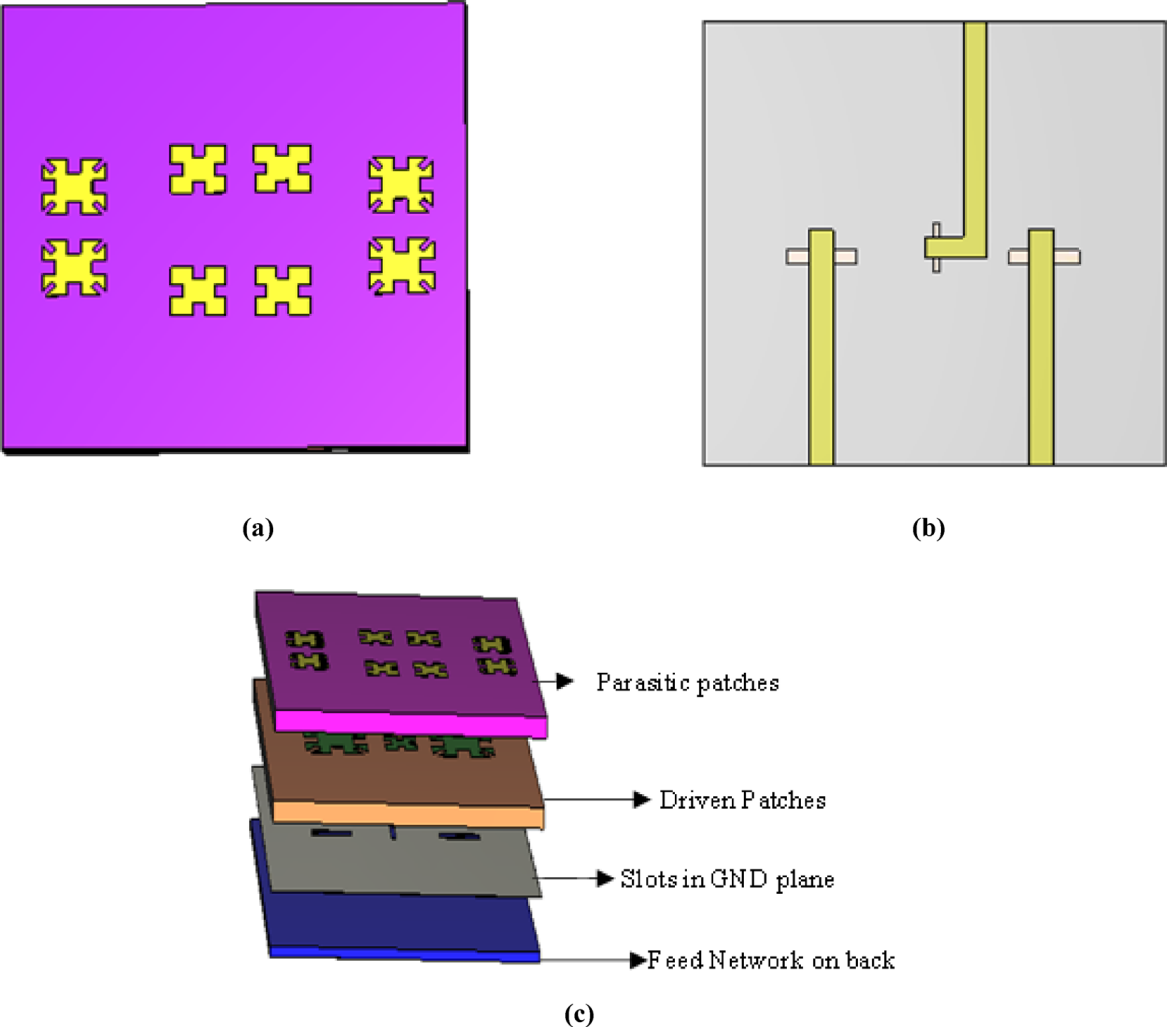
MIMO configuration. (**a**) Parasitic patches and driven patches. (**b**) Back of 3rd substrate. (**c**) 3D design of MIMO configuration.

To support multi-band operation and polarization diversity, a MIMO configuration is first constructed. The design consists of two vertically polarized X-band elements forming a two-port MIMO system, along with a centrally positioned, one horizontally polarized Ku-band element, as illustrated in Fig. 9a,b. Next, an additional Rogers 5880 substrate (with a permittivity of 2.2 and a thickness of 1.57 mm) is incorporated at the top of the structure. This substrate serves as a base for the four parasitic patches per band, effectively suppressing adjacent mode excitation. For compactness, each pair of driven patches for each operating band shares two parasitic patches. The 3D representation of the final MIMO configuration is shown in Fig. 9c.

**Fig. 10:**
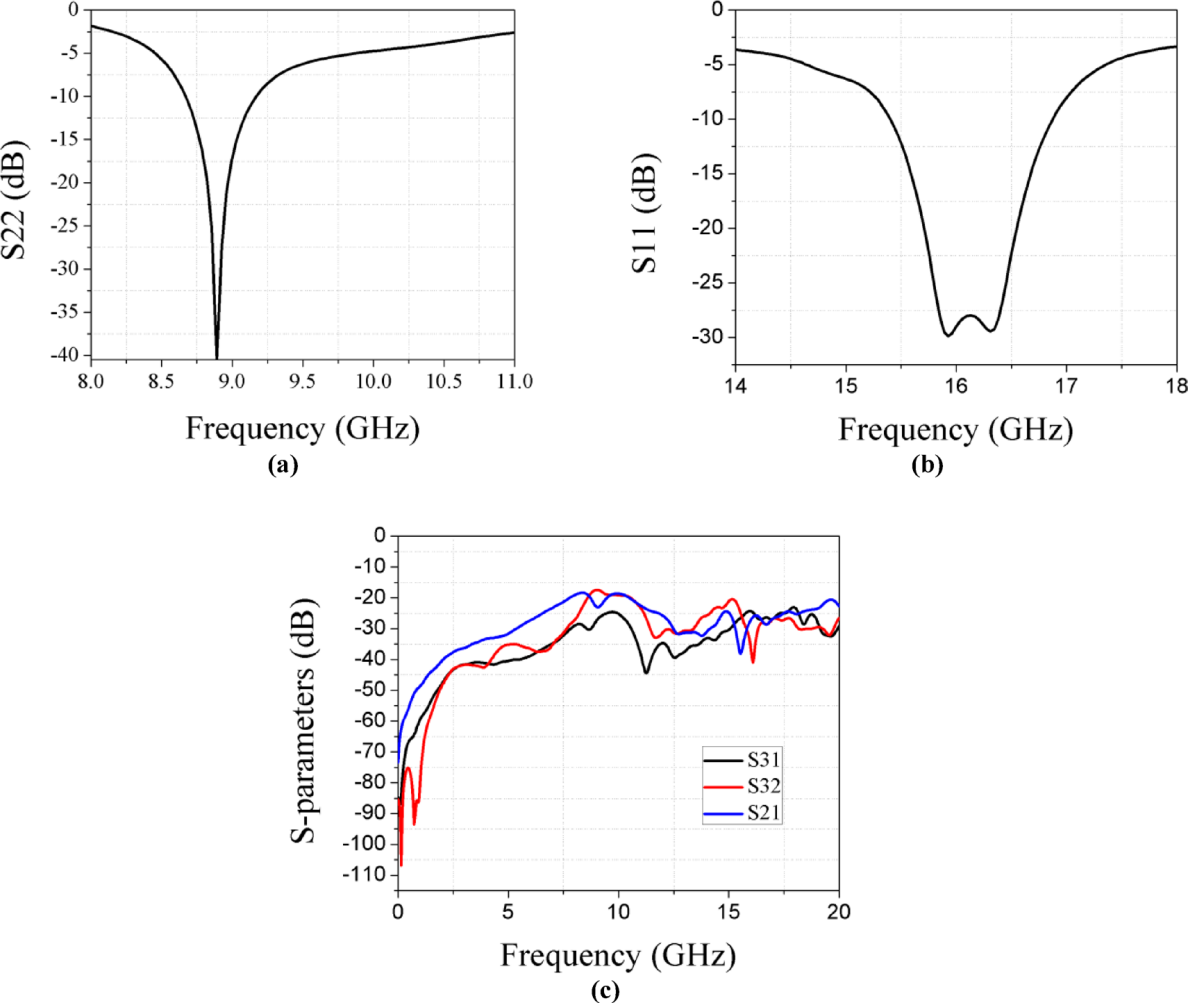
(**a**) Simulated results of X band in MIMO. (**b**) Simulated results of Ku band in MIMO. (**c**) Coupling between three ports in MIMO.

For the X-band MIMO configuration, the design operates at 9 GHz, with a gain of 6 dBi and bandwidth of 0.5 GHz. For the Ku-band, the design achieves optimal performance at 16 GHz, with a gain of 10.1 dBi and a bandwidth of 1.5 GHz, as shown in Fig. 10a,b respectively. Figure 10c demonstrates the coupling between elements.

### Antenna array configuration

A dual-band 1 × 4 antenna array is designed and tested to provide high gain and the desired radiation pattern, as depicted in Fig. 13d. The 1 × 4 antenna array is constructed in two stages: first, a 1 × 2 array is created and then replicated to form the 1 × 4 array.

#### Array 1 × 2 configuration

**Fig. 11:**
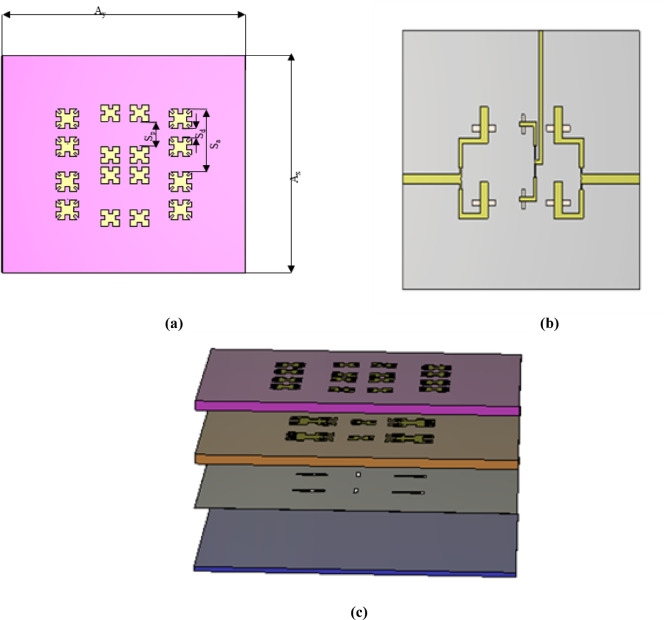
Array 1 × 2 configuration. (**a**) Parasitic patches only on top of 1st substrate. (**b**) Back of 3rd substrate Parasitic patches and driven patches. (**c**) 3D design of 1 × 2 array.

In Fig. 11a,b the previously mentioned MIMO design elements are arranged vertically to form a 1 × 2 array. The element spacing is selected as 13 mm to avoid the formation of high grating lobes. The total dimensions of the 1 × 2 array are Ax = 45.1 mm and Ay = 50.2 mm. The feed network is designed with varying widths to achieve different matching impedances. The network features three impedances (50, 70.7, and 100) ohms), arranged based on the Wilkinson power divider, as described by referring to (1) and (2) in^31^.

1 $$Z_{in} = Z_{1} \frac{{R_{L} + Jz_{1} \tan Bl}}{{z_{1} + jR_{L} \tan Bl }}$$

2 $$\begin{gathered} Z_{in} = \frac{{Z_{1}^{2} }}{{R_{L} }} \hfill \\ Z_{1} = \sqrt[2]{50 \times 100} = 70.7 \Omega \hfill \\ \end{gathered}$$

where ${Z}_{in}$ refer to input impedance, ${Z}_{1}$ refers to characteristic impedance, ${R}_{L}$ refers to load impedance, $B$ refers to propagation constant, *l* is the length of the transmission line.

**Fig. 12:**
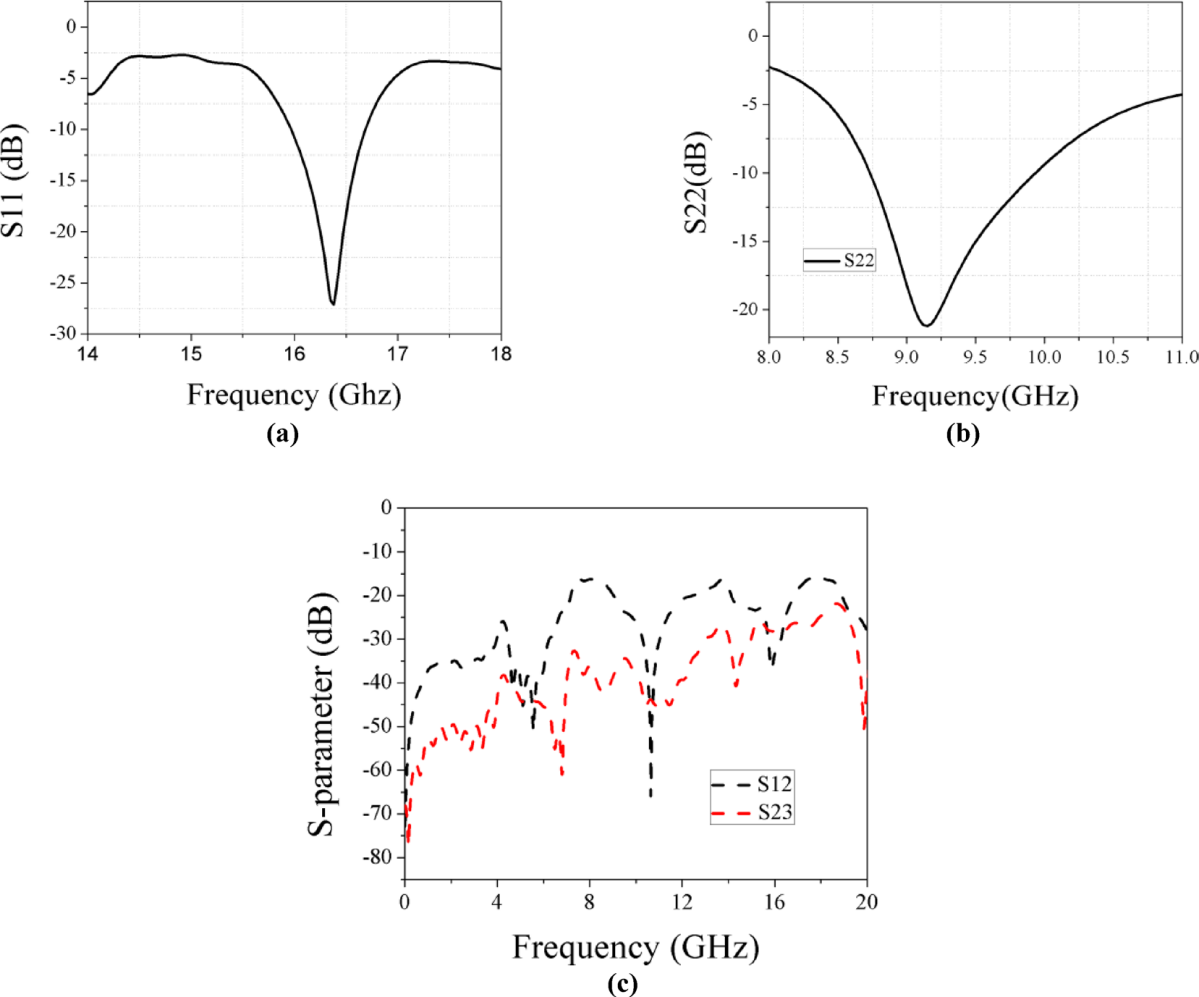
(**a**) Simulated result at Ku band in array 1 × 2. (**b**) Simulated result at X band in array 1 × 2. (**c**) Coupling parameters between three ports in array 1 × 2.

3D design of this array is illustrated in Fig. 11c. In Fig. 12a,b, the design achieves resonance at 9.15 GHz (X-band), with a bandwidth from 8.7 to 9.96 GHz and a gain of 6.3 dBi. At the Ku-band, the design achieves matching impedance at 16.4 GHz, with a gain of 11.4 dBi and a bandwidth from 15.9 to 16.67 GHz. Figure 12c demonstrates minimal coupling between elements.

#### Array 1 × 4 configuration

**Fig. 13:**
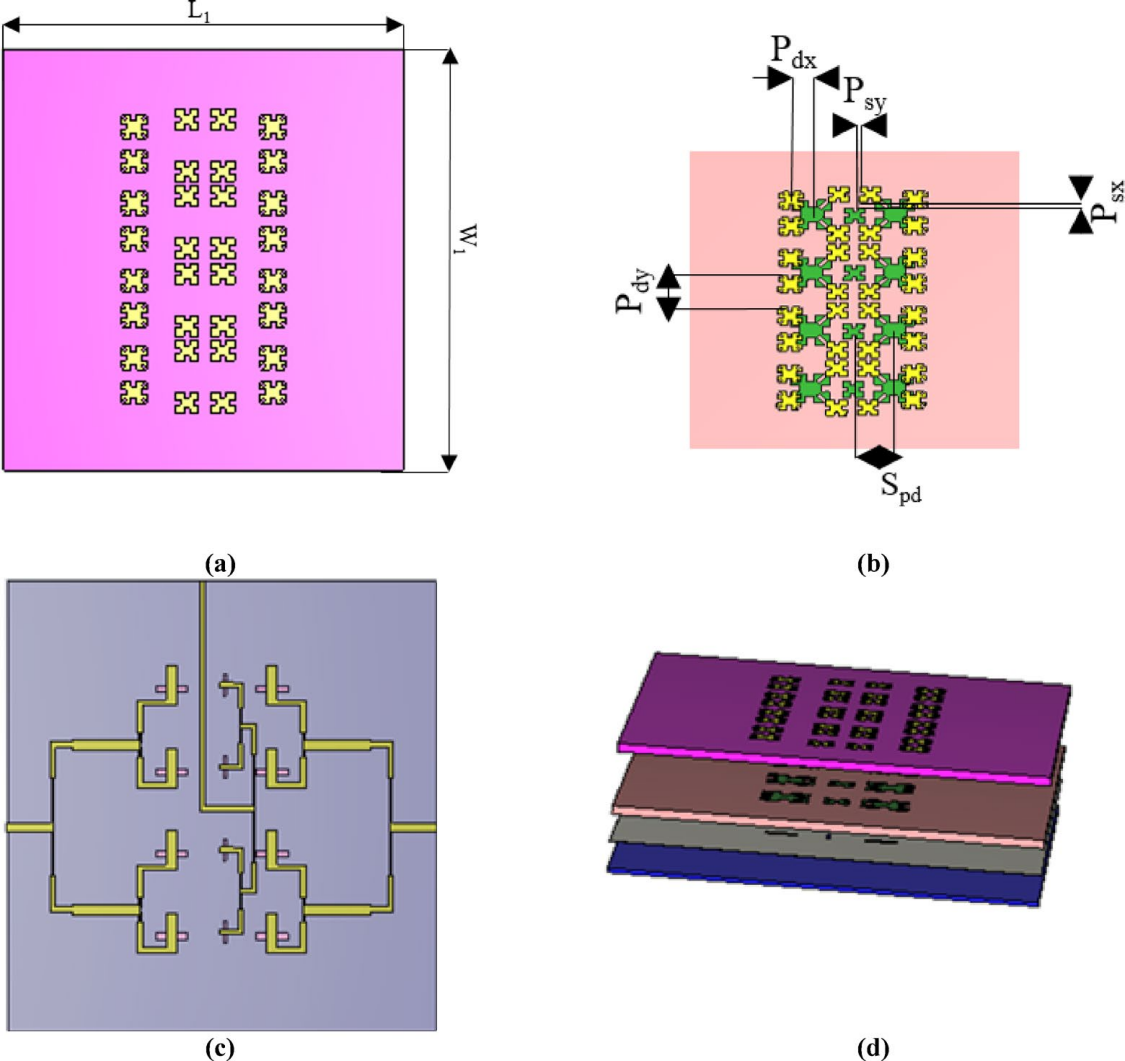
Final array configuration (**a**) Parasitic patches only on top of 1st substrate. (**b**)Parasitic patches and driven patches. (**c**) Back of array (**d**) 3D design of final array.

Final design is done by expanding the array to a 1 × 4 configuration with total dimension L1 = 65.1 mm and W1 = 71.2 mm. A gain of 11.17 dBi is achieved at the X-band, and a gain of 14.22 dBi is obtained at the Ku-band. In Fig. 13 from figure a to figure d the final 1 × 4 array is formed by vertically repeating the 1 × 2 array with the same element spacing. The feeding network is also expanded to support the four elements. Table 1 presents the array’s parameters.

**Table 1:** Parameters of final array.

Parameter	L_1_	W_1_	A_x_	A_y_	S_p_	S_d_	S_a_	S_pd_	P_sx_	P_sy_	P_dx_	P_dy_
Value (mm)	67.2	71.1	45	50.2	5.356	1.839	13	8.1	0.94	0.956	3.81	2.928

**Fig. 14:**
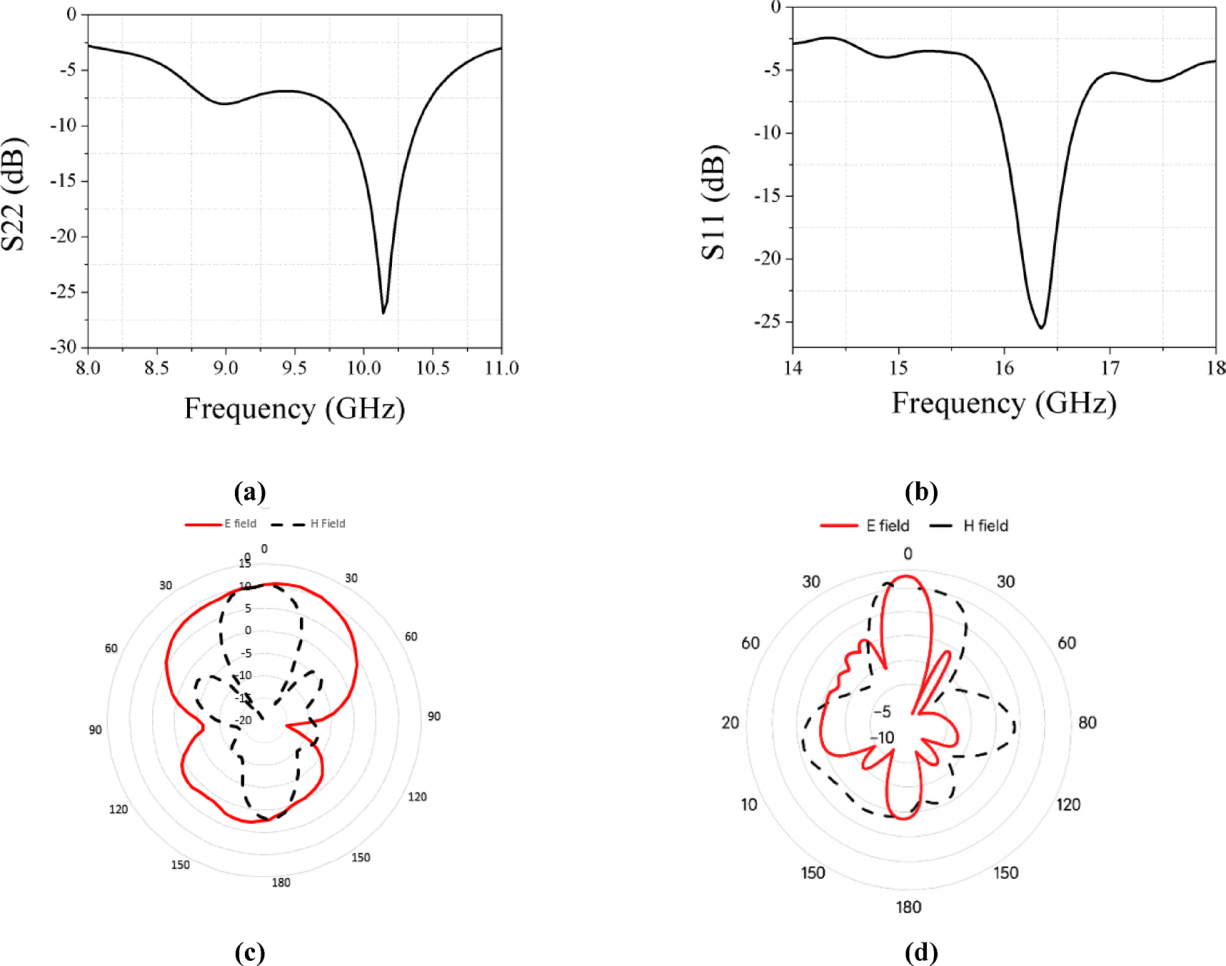
Final array configuration (**a**) Simulated results at X band in final array 1 × 4. (**b**) Simulated results at Ku band in final Array 1 × 4. (**c**) Simulated radiation pattern at X band in final array 1 × 4. (**d**) Simulated radiation pattern at Ku band in final array 1 × 4.

At the X-band, the simulated results show good matching at 10.14 GHz with a bandwidth of 0.52 GHz is shown in Fig. 14a, while at the Ku-band, the antenna operates at 16.35 GHz with a bandwidth of 0.67 GHz is shown in Fig. 14b. Good radiation pattern is achieved at X band and Ku band and are shown at Fig. 14c,d respectively.

## Fabrication and experimental results of array design

**Fig. 15:**
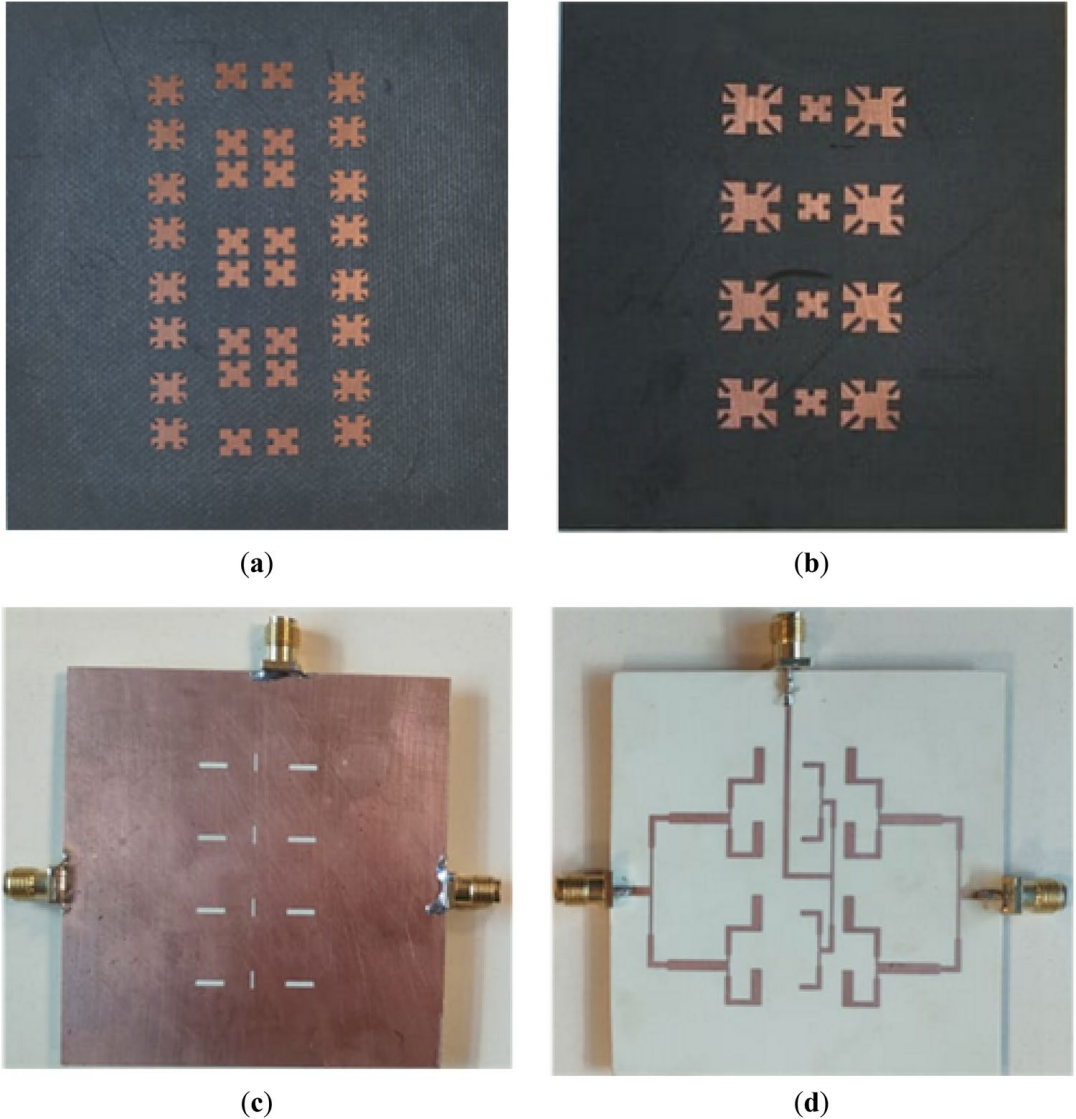
Fabricated final array (**a**) Parasitic patches only on top of 1st substrate (**b**) Driven patches on 2nd substrate. (**c**) Slots in ground Plane. (**d**) Feed network in ground plane.

The antenna array, as previously described in Fig. 13c, features three ports: the first and second ports, located on the right and left sides of the substrate, operate at the X-band, while the third port, at the top center, functions at the Ku-band. The array is fabricated using photolithography, and a photo of the fabricated array is shown in Fig. 15 from (a) to (d).

**Fig. 16:**
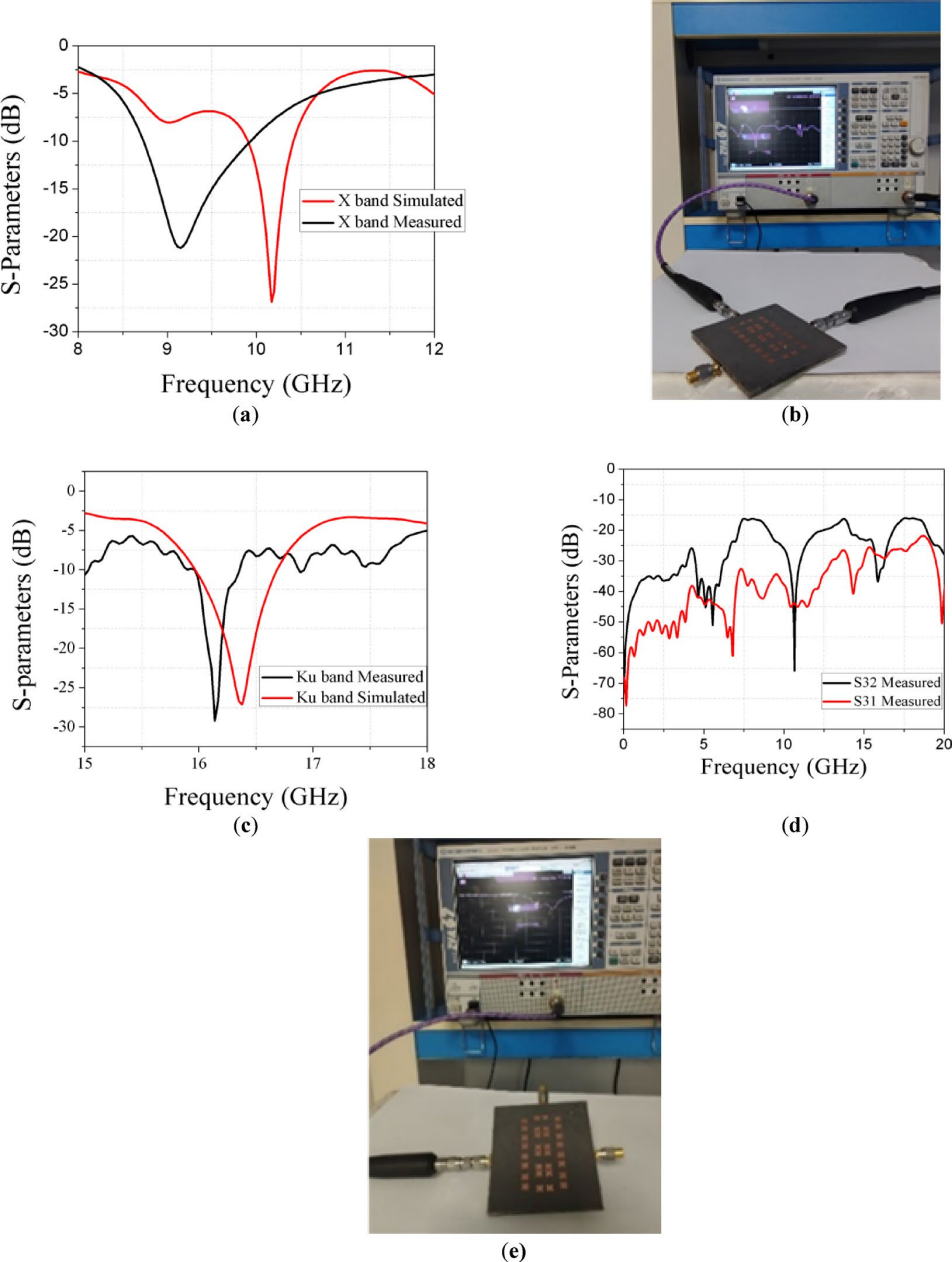
(**a**) Simulated and measured results of S11 at X band. (**b**) Setup of measuring S11 using VNA. (**c**) Simulated and measured results of S11 at KU band. (**d**) Measured coupling parameters between three ports. (**e**) Setup of measuring two ports using VNA.

The measured results are compared to the simulated results from CST 2021.In Fig. 16a shows comparison between simulated and measured results of S parameters at X band and Fig. 16c shows comparison between simulated and measured results of S-parameters at KU band. When measuring S11 one port is connected to VNA and other two ports are terminated by 50 Ohm matched load as shown in Fig. 16b. When measuring S21 or S31 two ports are connected and other port is terminated as shown in Fig. 16e. We found that there is some shift between simulated and measured results due to the fabrication alignment and soldering effects. Measured Coupling between ports is shown in Fig. 16d. It is found that for all port pairs, the isolation is below − 20 dB which is accepted.

**Fig. 17:**
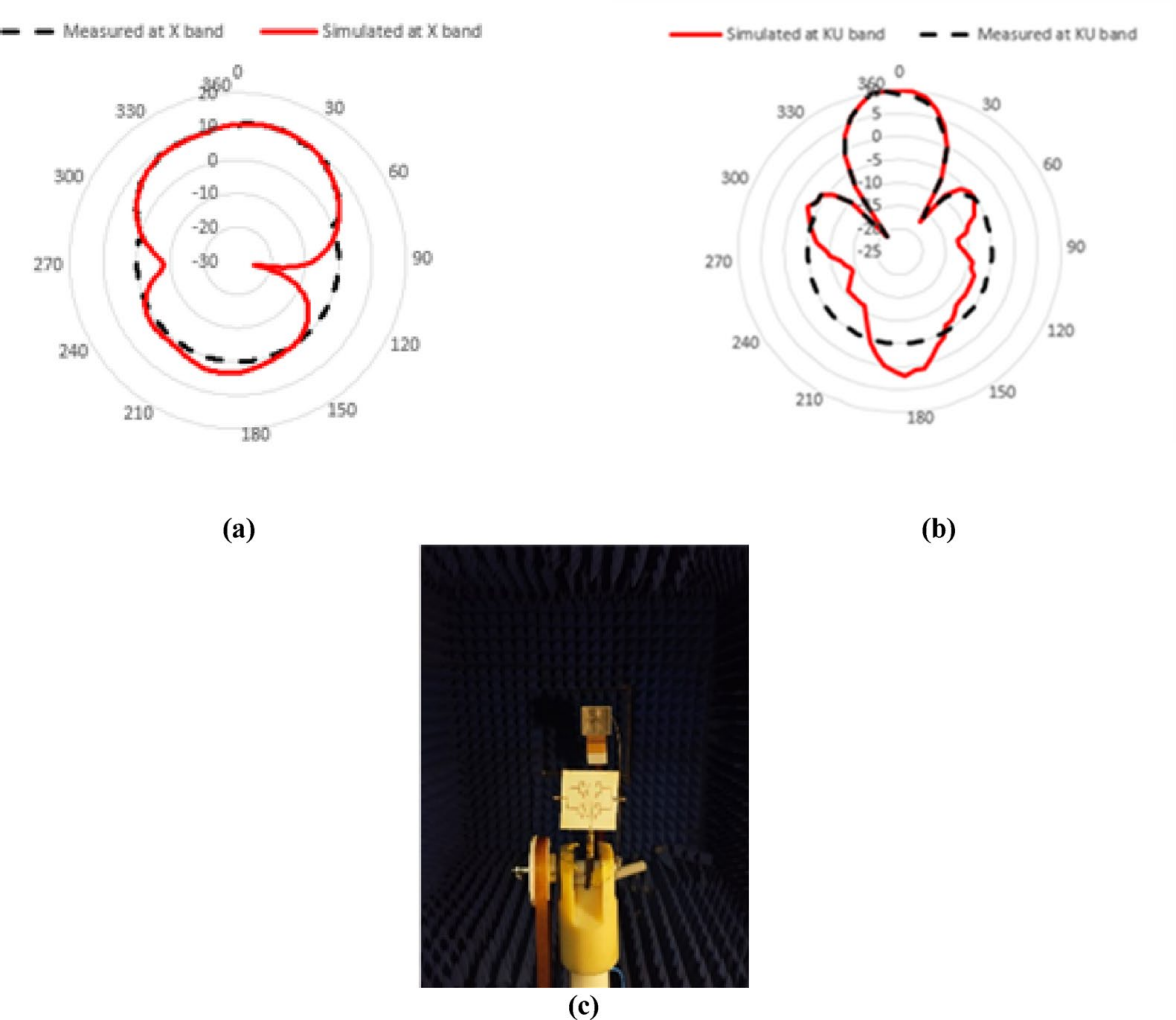
(**a**) Radiation pattern measurement at X band. (**b**) Radiation pattern measurement at KU band. (**c**) Setup of measuring radiation pattern.

Radiation patterns are measured at each frequency X and KU bands as shown in Fig. 17a and b respectively. Figure 17c shows photos of measuring radiation pattern of antenna array in anechoic chamber. Table 2 presents a comparison between the proposed design and recent state-of-the-art works. Compared to literature works, the proposed dual-band shared-aperture array antenna demonstrates a compact structure and competitive performance across both X- and Ku-bands. Unlike^32^, which uses a large 33-element grid array with size of 11.688 λ × 1.44 λ working at 24  GHz has a bandwidth of 20.8% and a gain of 14.2  dBi, the proposed design achieves dual-band functionality with a significantly reduced aperture size (1.955  λ × 2.138  λ) while maintaining high gain (11.12  dBi at 9  GHz and 14.22  dBi at 16.1 GHz). In contrast to^33^, which targets lower frequencies S- and L-bands and employs a bulkier 4 × 4 cavity slot array with moderate gains (7.3 dBi and 13 dBi), the present work supports higher-frequency operation with improved spatial efficiency. Similarly^34^, presents a finite 8 × 8 array for Ku-band only, with a lower gain of 5.1 dBi and no reported aperture size, whereas the proposed antenna covers both X and Ku bands with better polarization control (vertical and horizontal). The dual-band AMC-backed structure in^35^ covers slightly overlapping Ku-band ranges but requires a larger area (3.339 λ × 3.339 λ) and more complexity via a 6 × 6 AMC layer, achieving comparable gain but lower integration efficiency. Moreover^36^, offers a compact 2 × 2 array at 28 GHz with modest bandwidths (1.43%, 2.29%), which are lower than those achieved in the present work. The proposed design distinguishes itself through its dual-band capability, shared-aperture compactness**,** high gain**,** cross-polarization suppression below − 10 dB with orthogonal polarization diversity, offering an efficient and structurally simple alternative to more complex and band-limited designs.

**Table 2:** Comparison between the paper and other papers.

References	Frequency (GHz)	Electrical dimension (λ)	BW (%)	Gain (dBi)	SLL (dB)	No of elements	Configuration	*Pol
^32^	(24)	11.688 λ × 1.44 λ	20.8%	14.2	− 16.3	33 Radiating Elements	Grid array	Linear
^33^	(3.4–3.8) (0.69–0.96)	1.394 λ × 1.394 λ	3.4% 3.8%	7.3 13	< -10	4 × 4	Shared aperture Cavity Slot Antenna-in-Package	Linear
^34^	(28–30.5)	NA	8.5%	5.1	NA	8 × 8	finite array with 8 × 8 proposed unit cells	Circular
^35^	(25.36–26.03) (26.75–28.81)	3.339 λ × 3.339 λ	2.7% 7.6%	11.8–13.1	< − 10	1 × 4 anay + 6*6 AMC	Adding AMC	linear
^36^	(28)	3.45 λ × 3.45 λ	1.43% 2.29%	12.5	NA	2 × 2	Short Ring + Slot	Linear
This paper	(9) (16.1)	1.955 λ × 2.138 λ	5.7% 4.1875%	11.12 14.22	< − 10	1 × 4 array	Shared Aperture array antenna	Vertical Horizontal

*Pol: Polarization.

The key contribution of this paper lies not merely in its compactness, but in achieving practical integration of dual-band functionality (X band, and Ku band), polarization diversity, and high radiation performance. This distinct combination of features represents a meaningful advancement over existing solutions, which often involve trade-offs in complexity and efficiency. The proposed design achieves high gains of approximately 11 dBi at 9 GHz and 14.2 dBi at 16.1 GHz. Compared to conventional designs that rely on large apertures or are limited to single-band operation, this antenna provides an efficient, dual-band solution with enhanced polarization capability. It is specifically tailored for GB-SAR systems, offering improved spatial resolution and imaging flexibility for critical environmental monitoring applications such as landslide detection and terrain deformation analysis.

## Conclusion

The proposed compact microstrip antenna array operates at dual bands, X and Ku, with different polarizations for remote sensing applications. It consists of four driven elements, each electromagnetically coupled to four parasitic patches for bandwidth enhancement. Two ports support vertical polarization in the X-band with a 0.52 GHz bandwidth, while the third port supports horizontal polarization in the Ku-band with a 0.67 GHz bandwidth. Good bandwidth is achieved with a compact shape and a simple feed network. The antenna achieves a gain of 11.17 dBi at the X-band and 14.22 dBi at the Ku-band, which is suitable for this application.

## Data Availability

All data generated or analyzed during this study are included in the published article. Dr Anwer is corresponding author who who can be contacted in order to access these data.

## References

[1] Moreira, A. et al. A tutorial on synthetic aperture radar. *IEEE Geosci. Remote sens. Mag.* **1**(1), 6–43 (2013).

[2] Koukiou, G. SAR features and techniques for urban planning—A review. *Remote Sens.* **16**(11), 1923 (2024).

[3] Hanssen, Ramon, F. et al. Synthetic aperture radar for geosciences. *IEEE Geosci. Remote Sens. Mag.* **1**, 7–23 (2024).

[4] El-Hameed, A. S. A. & Sato, M. Antenna array for Ku-band MIMO GB-SAR. *IEEE Access* **9**, 29565 (2021).

[5] Liu, J., Yu, X. & Zhou, H. Dual-band dual-polarized waveguide slot antenna array for SAR applications. *IEEE Antennas Wireless Propag. Lett.* (2020).

[6] Vetrella, A., Lanari, R., Scarfò, M. & Budillon, A. W-Band MIMO GB-SAR for bridge testing/monitoring. *Electronics* **10**, 1–18 (2021).

[7] Qin, F. et al. A simple low-cost shared-aperture dual-band dual-polarized high-gain antenna for synthetic aperture radars. *IEEE Trans. Antennas Propag.* **64**, 2914–2922 (2016).

[8] Shafai, L., Chamma, W., Seguin, G. & Sultan, N. Dual-band dual-polarized microstrip antennas for SAR applications. *IEEE Antennas Propag. Soc. Int. Symp.* **3**, 1866–1869 (1997).

[9] Shafai, L. L., Chamma, W. A., Barakat, M., Strickland, P. C. & Seguin, G. Dual-band dual-polarized perforated microstrip antennas for SAR applications. *IEEE Trans. Antennas Propag.* **48**, 58–66 (2000).

[10] Pozar, M. & Targonski, S. D. A shared-aperture dual-band dual polarized microstrip array. *IEEE Trans. Antennas Propag.* **49**, 150–157 (2001).

[11] Vetharatnam, G., Kuan, C. B. & Teik, C. H. Combined feed network for a shared-aperture dual-band dual. *IEEE Antennas Wireless Propag. Lett.* **4**, 297–299 (2005).

[12] Zhong, S.-S., Sun, Z., Kong, L.-B., Wang, C. W. & Jin, M.-P. Tri-band dual-polarization shared-aperture microstrip array for SAR applications. *IEEE Trans. Antennas Propag.* **60**, 4157–4165 (2012).

[13] Kaboli, M., Abrishamian, M. S., Aboutorab, S. M. & Mirtaheri, S. A. High-isolation XX-polar antenna. *IEEE Trans. Antennas Propag.* **60**, 4046–4055 (2012).

[14] Qu, X., Zhong, S. S., Zhang, Y. M. & Wang, W. Design of an S/X dual-band dual-polarised microstrip antenna array for SAR applications. *IET Microw. Antennas Propag.* **1**, 513–517 (2007).

[15] Izumi, Y., Saito, R., Abd El-Hameed, A. S., Fujiwara, J. & Sato, M. *Evaluation of Atmospheric Phase Screen in 79 GHz MIMO Radar Interferometry*. *IGARSS*, Pasandena, USA, (2023).

[16] Abd El-Hameed, A. S., Ouf, E. G., Elboushi, A., Seliem, A. G. & Izumi, Y. An improved performance radar sensor for K-band automotive radars. *Sensors* **23**(16), 7070 (2023). 37631607 10.3390/s23167070 PMC10459370

[17] Elnady, S. M., Abd El-Hameed, A. S., Ouf, E. G. Innovative K-band slot antenna array for radar applications. J. Electric. Syst. Inf. Technol. **11**, 34 (2024). 10.1186/s43067-024-00159-9.

[18] Kong, L. & Xu, X. A compact dual-band dual-polarized microstrip antenna array for MIMO-SAR applications. *IEEE Trans. Antennas Propag.* **66**, 2374–2381 (2018).

[19] Fujimoto, T. & Fukahori, S. Broadband dual-band stacked square microstrip antenna with shorting plates and slits. IET Microw. Antennas Propag. **6**, 1443–1450 (2012).

[20] Lu, J.-H. Broadband dual-frequency operation of circular patch antennas and arrays with a pair of L-shaped slots. *IEEE Trans. Antennas Propag.* **51**, 1018–1023 (2003).

[21] Shavit, R., Tzur, Y. & Spirtus, D. Design of a new dual-frequency and dual-polarization microstrip element. *IEEE Trans. Antennas Propag.* **51**, 1443–1451 (2003).

[22] Meng, F. & Sharma, S. K. A dual-band high-gain resonant cavity antenna with a single layer superstrate. *IEEE Trans. Antennas Propag.* **63**, 2320–2325 (2015).

[23] Nunna, B. A. & Kothapudi, V. K. Design and analysis of 4×4 X-band conformal antenna array with corporate series feeding for spaceborne SAR applications. in *Proceedings of the Second International Conference on Computational Electronics for Wireless Communications* 193–204 (2023).

[24] Jiang, Z. N., Zheng, Y., Xuan, X. F. & Nie, N. Y. A novel ultra-wideband wide-angle scanning sparse array antenna using genetic algorithm. *Appl. Comput. Electromagn. Soc. J.* **38**, 100–108. 10.13052/2023.ACES.J.380204 (2023).

[25] Sepehripour, F., Shafiei Alavijeh, A., Fakharzadeh, M. & Khavasi, A. A broadband and compact millimeter-wave imaging system based on synthetic aperture radar. *arXiv preprint *arXiv:2205.14707, 2205.14707, (2022).

[26] Wang, Y. & Du, Z. Dual-polarized dual-band microstrip antenna with similar-shaped radiation pattern. *IEEE Trans. Antennas Propag.* **63**, 5923–5928 (2015).

[27] Nasimuddin, N. & Chen, Z. N. Wideband microstrip antennas with sandwich substrate. IET Microw. Antennas Propag. **2**, 538–546 (2008).

[28] Nasimuddin, Esselle, K.P. & Verma. A.K. Wideband circularly polarized stacked microstrip Antennas. IEEE Trans. Antennas Propag. **56**, 578–581 (2008).

[29] Nasimuddin, & Chen, Z. N. Wideband multilayered microstrip antennas fed by coplanar waveguide-loop with and without via combinations. *IET Microwaves Antennas Propag.* **3**(1), 85–91 (2009).

[30] Balanis, C. A. *Antenna Theory: Analysis and Design* 3rd edn. (Wiley-Interscience, 2005).

[31] Pozar, D. M. Power dividers and directional couplers. in *Microwave Engineering*, 4th edn. Wiley. New York, NY, USA, 0–75 (2012).

[32] Arumugam, S., Palaniswamy, S. K. & Manoharan, S. High gain wide band grid array antenna for short range radar and vehicle-to-satellite communications. *AEU Int. J. Electron. Commun.* **147**, 154157 (2022).

[33] Niu, W., Sun, B., Zhou, G. & Lan, Z. Dual-band aperture shared antenna array with decreased radiation pattern distortion. *IEEE Trans. Antennas Propag.* **70**(7), 6048–6053. 10.1109/TAP.2022.3161267 (2022).

[34] Zhang, L. & Yin, J. Ka-band wide-angle scanning phased array with dual circular polarization. *Electronics* **13**(12), 2238. 10.3390/electronics13122238 (2024).

[35] Ibrahim, A. A. et al. Slotted antenna array with enhanced radiation characteristics for 5G 28 GHz communications. *Electronics* **11**(17), 2664. 10.3390/electronics11172664 (2022).

[36] Kim, Y.-J., Kim, Y.-B. & Lee, H. L. mm Wave high gain planar h-shaped shorted ring antenna array. *Sensors* **20**(18), 5168. 10.3390/s20185168 (2020). 32927814 10.3390/s20185168 PMC7571215

